# The psychological impact of paediatric burn injuries: a systematic review

**DOI:** 10.1186/s12889-021-12296-1

**Published:** 2021-12-14

**Authors:** Alix Woolard, Nicole T. M. Hill, Matthew McQueen, Lisa Martin, Helen Milroy, Fiona M. Wood, Indijah Bullman, Ashleigh Lin

**Affiliations:** 1 Telethon Kids Institute, Perth Children’s Hospital, 15 Hospital Avenue, Nedlands, Australia; 2 The University of Western Australia, Perth, Australia; 3 Fiona Wood Foundation, Perth, Australia; Child and Adolescent Health Service, Perth Children’s Hospital, University of Western Australia, Perth, Western Australia Australia; 4 Fiona Wood Foundation, Perth, Australia

**Keywords:** Burn, Pediatric, Psychological outcomes, Psychopathology

## Abstract

**Objective:**

To review and synthesise qualitative literature regarding the psychological outcomes following paediatric burn injuries, and to determine if children and adolescents who experience a burn injury have elevated risk of psychopathology following the injury.

**Design:**

Systematic review of quantitative and qualitative studies.

**Data sources:**

Informit health, Medline, Embase, and PsycINFO were searched from January 2010 to December 2020.

**Data extraction and synthesis:**

Two reviewers screened articles, and one reviewer extracted data (with cross-checking from another reviewer) from the included studies and assessed quality using an established tool. Narrative synthesis was used to synthesise the findings from the quantitative studies, and thematic synthesis was used to synthesise the findings of included qualitative studies.

**Results:**

Searches yielded 1240 unique titles, with 130 retained for full-text screening. Forty-five studies from 17 countries were included. The psychological outcomes included in the studies were mental health diagnoses, medication for mental illness, depression, anxiety, stress, fear, post-traumatic stress, post-traumatic growth, emotional issues, self-harm, self-esteem, self-concept, stigmatisation, quality of life, level of disability, resilience, coping, and suicidality.

**Conclusions:**

Our findings highlight paediatric burn patients as a particularly vulnerable population following a burn injury. Studies suggest elevated anxiety and traumatic stress symptoms, and higher rates of psychopathology in the long-term. Further research is recommended to determine the psychological outcomes in the other mental health domains highlighted in this review, as findings were mixed.

Clinical care teams responsible for the aftercare of burn patients should involve psychological support for the children and families to improve outcomes.

## Introduction

Burns are one of the most severe injuries that a child can experience and are a common cause of emergency presentations. The most recent Australian annual report showed 5430 cases of hospitalisation for burns, the majority of which were young children (aged 0–4) [1]. Paediatric burn management and medical aftercare has made significant advances in recent decades (for a review, see; [2]), such that current survival rates are high for even very serious burns [3, 4]. Historically, burn aftercare has focused on physical healing, in recent years, however, research has uncovered the poor psychological outcomes in children who experience burns, including a minor burn injury [5]. This is because burn injuries do not only have a serious physical impact on a child, they also seriously impact the psychological and emotional wellbeing of the child and their family.

Burn injuries are painful, both physically and mentally. This is especially the case for young children who may not understand that procedural pain (e.g., dressing changes) is a necessary component of recovery [6]. In fact, early burn studies provided some of the founding research for the original clinical outline of posttraumatic stress disorder (PTSD) in the Diagnostic and Statistical Manual of Mental Disorders-III [7, 8]. The event in which the injury occurred is often traumatising [9], and hospitalisation can be scary for children and often involves separation from family or peers, which is traumatic in itself [10]. Further, the immediate and subsequent wound care following a burn injury is not only physically painful, but also invasive and pervasive [11]. Scarring is also common for a burn wound, despite medical advances, which requires long-term medical care and can also contribute to poor psychological outcomes [9].

Given the severity and prevalence of paediatric burn injuries, as well as the accompanying trauma during and after a burn, there is a need to address the psychological after-care of paediatric burns. Symptoms of PTSD are often reported in children who have experienced a burn injury [12–14]. Other studies have also found that post-burn, individuals can report high rates of acute stress disorder [15], anxiety, and depression [9].

It is important when attempting to address clinical outcomes, such as the psychological after-care of paediatric burns, to first review the available evidence on the subject and make evidence-based evaluations. The existing reviews of paediatric burn outcomes reveal that the majority of children who have experienced paediatric burn injuries often go on to experience long-term psychopathology [16–18]. There are studies that contradict these findings, with reports of children adjusting well after this injury [19]. However, the most recent review on psychopathology following paediatric burns, conducted 8 years ago, found that the overwhelming majority of studies report short- and long-term psychopathology in both children and their parents after a burn injury [10]. This review highlighted that the majority of children who have experienced a burn injury go on to display some sort of distressed behaviour, either internalising (such as anxiety and withdrawal) or externalising (such as aggression and opposition) behaviours [10]. The findings highlight the importance of psychological aftercare and how advances in mental health care following a burn injury had greatly improved between 2000 and 2012, giving clinicians a better understanding of the broader needs of those who have experienced paediatric burns [10].

There is a need for an updated review of the work by Bakker et al. [10] of the current research on the psychological impact and outcomes of paediatric burns. Clinicians need to be aware of the current research on the psychological outcome of paediatric burns survivors to be able to target areas of need – which extend beyond treating the physical burn. The aim of this current review was to search, review, and evaluate the current research that has been conducted on the psychological impact on children’s mental health following paediatric burn injury. The primary objective of this study was to examine the research (conducted in the last 10 years) that exists on the psychological impact on children’s mental health following a paediatric burn injury. We hypothesised that children or adolescents who sustained a burn injury would be more likely to go on to experience psychopathology than those who had not experienced a burn injury.

## Method

We conducted this review following the Preferred Reporting Items for Systematic Reviews and Meta-Analyses (PRISMA) statement guidelines [20]. Our review methodology was registered with the PROSPERO international prospective register of systematic reviews (CRD42020215553, National Institute for Health Research, n.d.).

### Search strategy

Electronic databases Informit health, Medline, Embase, and PsycINFO were searched on the 19th of October 2020, for English-language empirical peer-reviewed articles published in the last 10 years (2010–2021). Our search terms included: ‘children’, ‘chil*’, ‘paediatric’, ‘pediatric’, ‘youth’, ‘young’, ‘adolesc*’, AND ‘burn*’, ‘thermal*’ AND ‘psycholog*’, ‘psychopathology’, ‘mental health’, ‘depression’, ‘anxiety’. These terms were derived from our research question, similar published reviews, and through consultation with experts in the field. Additional articles were identified through comprehensive hand searches of the reference lists of included articles and Google Scholar searches. Two authors (AW, NTMH) conducted the initial screening of abstract and titles, and the full-text screen of potential articles. Any discrepancies were resolved by two authors (AW, AL).

### Inclusion and exclusion criteria

Articles were included if they involved: 1) data on the psychological impact or psychological outcome of paediatric burn injury, 2) mean age of children in the study was under 18 years of age at the time of the burn injury, 3) the injury involved a burn that required hospitalisation (including emergency presentation). Psychological outcomes were reported from parent-report or caregiver-report questionnaires, child self-report questionnaires, or from observational assessment. Cross-sectional or cohort studies were eligible if the psychological outcomes were assessed after the burn injury. Randomised control trials (RCTs) were eligible if psychological measures were assessed at baseline (pre-intervention). Eligible control groups included child or adolescent populations that did not experience a burn injury.

Articles were excluded if they; 1) only included physiological or functional outcomes of the child only, 2) articles with adult populations, 3) papers not peer-reviewed (e.g. grey literature and book chapters), 4) intervention studies that focussed on a drug or behavioural treatment of the burn injury and did not measure psychological variables at baseline, 5) psychological outcomes related to parents of the child only, 6) burn injury arose from self-harm, potentially indicating prior psychopathology, and 7) burn injury was reported as the result of intentional harm (such as maltreatment by a caregiver). Non-English language studies were included and translated using Google translate. The first author contacted authors for further details (e.g., unpublished data, further information from abstracts) on grey literature to avoid publication bias.

### Data collection, risk of bias and quality assessment

Data were extracted using the software Covidence [21], which has a data extraction template. Results were reported according to the PRISMA statement (Liberati et al., 2009). Study quality was assessed using the National Heart, Lung and Blood Institute quality assessment tool for observational, cohort and cross-sectional studies, with the addition of the following criteria relevant to this review: (1) the study involved a comparison groups: a paediatric burn group and a control non-burn group; (2) reliable and validated psychological outcome measures were used; (3) a statistical power analysis was conducted to determine optimal sample size to find differences between groups; (4) appropriate statistical analyses were conducted to determine differences between groups; and (5) the study used a prospective design rather than a cross-sectional design [22]. The quality assessment tool provides a rating for each study based on 14 criteria relating to the study population selection, blinding, confounding, outcome measures, and missing data or attrition. One reviewer (AW) rated each study using this tool, another author cross-checked (NTMH), and any discrepancies were sent to a third independent reviewer (AL) for deliberation.

## Results

### Study selection and characteristics

**Fig. 1:**
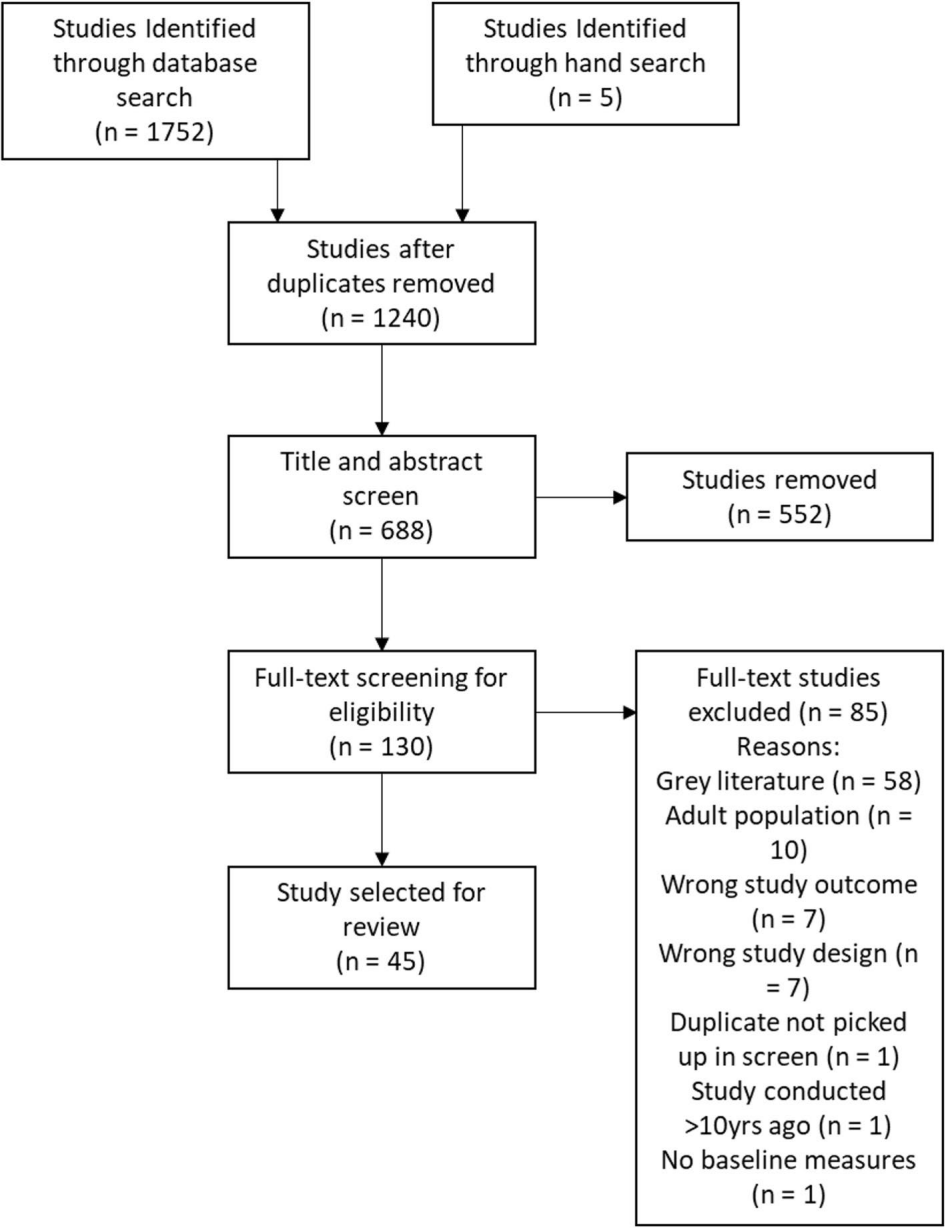
PRISMA flowchart of study selection

Forty-five studies we included in the review. Study selection was summarized using the PRISMA chart in Fig. 1. Study characteristics are outlined in Table 1. The studies comprised 32 mixed method, 10 interventions, and four qualitative. The age ranges of the children at the time of the burn injury varied in each study and ranged from 0 to 18 years of age. The settings of the studies included the United States (*n* = 17), Australia (*n* = 12), Sweden (*n* = 3), Canada (*n* = 2), the United Kingdom (*n* = 1), South Africa (n = 1), Nicaragua (n = 1), Mexico (n = 1), Iran (n = 1), Poland (n = 1), Switzerland (n = 2), Finland (n = 1), Brazil (n = 1), Spain (n = 1), the Netherlands (*N* = 1) and Turkey (n = 1). The psychological outcomes included in the studies were mental health diagnoses, medication for mental illness, depression, anxiety, stress, fear, post-traumatic stress, post-traumatic growth, emotional issues, self-harm, self-esteem, self-concept, stigmatisation, quality of life, level of disability, resilience, coping, and suicidality. Eighty-eight per cent of studies used validated and standardised assessments.

**Table 1:** Results of the quantitative studies

Randomised Controlled Trials							
Study authors, year, and country	Participant Description	Psychological outcomes/measures	Time since burn	Control/Comparator Group Outcomes	Treatment Group Outcomes	Other relevant findings	Effect size
Brown 2014, Australia	Range = 4–13 years *N* = 75	Anxiety and stress -VAS-A Child Trauma – CTSQ	None	VAS-A = 2.76	VAS-A = 2.60	–	Not reported
Chester 2018, Australia	Range = 4–16 years *N* = 64 Treatment group received hypnotherapy	Procedural anxiety - VAS-A PTSD - YCPC; PTSD symptoms only reported 3 months post intervention	None	VAS-A = 2.14 (3-months post-intervention)	VAS-A = 2.43 (3-months post-intervention)	–	Not reported
Hyland 2015, Australia	Range = 0–16 years *N* = 100 Treatment group received CLT	Children’s fear scale, parent/staff assessment of anxiety - VAS-A	0–5 days	Baseline anxiety = 2.0 Post intervention anxiety = 2.9	Baseline anxiety = 2.0 Post-intervention anxiety = 1.7	–	Not reported
Jeffs 2014, United States	Range = 10–17 years *N* = 28	Anxiety - STAIC, Procedural Behavior Checklist	None	STAIC state score = 31.5 (SD = 8.7) STAIC trait score = 34 (SD = 6.5)	STAIC state score = 31.5 (SD = 8.7) STAIC trait score = 34 (SD = 6.5)	Trait anxiety was negatively correlated with distraction engagement, indicating that as trait anxiety increased, engagement with distraction decreased.	Not reported
Maskell 2014, Australia	Range = 8–16 Mean = intervention = 12.23 (SD = 1.97), control = 13.31 (SD = 2.22) *N* = 63	QoL - PedsQL, psychological adjustment and psychopathology – SDQ self-concept - P-H SCS	Not reported beyond “burn past the acute stage of healing”.	PedsQL = 77.97 (SD = 15.66) SDQ = 12.57 (SD = 5.2) P-H SCS = 49.43 (SD = 10.51)	PedsQL = 80.12 (SD = 14.53) SDQ = 12.03 (SD = 6.31) P-H SCS = 50.82 (SD = 9.80)	Age and gender had significant interaction effects, with adolescent girls responding well to treatment.	Not reported
Parlak Gurol 2010, Other: Turkey	Range = 12–18 years N = 63 Treatment group received massage therapy	Anxiety - STAI	Mean = 3 days	STAI = 45.96 (SD = 7.02)	STAI = 46.71 (SD = 8.40)	Significance of difference between groups was *p* > .001	Not reported
Rezazadeh 2020, Other: Iran	Range = 6–12 years *N* = 60 Treatment groups received a music or painting based intervention	Anxiety (Spence Children’s Anxiety Scale), depression (Maria Kovacs Children’s Depression Inventory)	Not reported – intervention completed before discharge from hospital	Pre-intervention anxiety = 77.4 ± 13.8, depression = 28.9 ± 5.4	Music group pre-intervention anxiety = 84.8 ± 6.8, depression = 32.8 ± 5.4 Painting pre-intervention anxiety = 90.4 ± 5.4, depression = 38.7 ± 3.4	–	Not reported
Sharp 2010, United States	Mean = 6–7 years *N* = 363 Treatment group received propranolol	ASD - acute stress disorder symptom checklist, psychologist interview	< 30 days	5% required treatment for ASD	8% required treatment for ASD	No statistical difference was observed between groups	
Stoddard 2011, United States	Range: 6–20 Mean = 12.35 years (SD = 3.7) *N* = 26	PTSD symptoms -Diagnostic Interview Schedule for Children and Adolescents, Children’s PTSD Inventory, Depression - CDI	Not reported	Baseline CDI = 10.7 Baseline parent-rated PTSD score = 7.0 Baseline child-rated PTSD score = 8.8	Baseline CDI = 6.5 Baseline parent-rated PTSD score = 10.2 Baseline child-rated PTSD score = 10	CDI difference was the only significant difference between groups (*p* = .076)	Not reported
Cohort Studies							
Study authors, year, and country	Participant Description	Psychological outcomes/measures	Time since burn		Results	Effect size	
Boles 2018, Canada	Range = 1–17 years *N* = 47	Depression (identified via hospital records)	1–10 years		15 participants (32%) had symptoms of depression with or without suicidal ideation at time of injury	None reported	
Bushroe 2018, United States	Range = 0–18 years *N* = 2208	Mental health diagnoses, psychotropic medication prescription, rates of diagnoses or medication pre- and post-injury	1 year		20.7% of the population were children who sustained burn injuries, The rate of children with any mental health-related visit increased postinjury to 156.7 per 1000 person-years from 95.9 per 1000 person-years pre-injury, resulting in a RR of 1.63 (95% CI 1.39, 1.92). After adjusting for race and ethnicity, the impact on mental health diagnoses for children with burn injuries was most in-creased for young patients, ages 0–4 years (aRR: age 0–4 years, 8.56, 95% CI 3.30–22.18), compared with the older children (aRR age 10–14 years, 1.02, 95% CI 0.26–4.07). Age at injury was most associated with mental health post injury- younger patients (0–4) were more likely to have increased mental health problems	Any mental health diagnosis = **0.90 Adjustment disorders = **5.41 Anxiety disorders = **1.13 ADHD = **0.61 Bipolar disorders = **1.13 Disruptive behavior disorders = **1.09 Eating disorders = **0.67 Learning/cognitive disorders = **1.54 Nonbipolar depressive disorders = **0.60 Pervasive developmental disorders = **0.60 Sleep disorders = **1.28 Substance use disorders = **0.23 Other disorders = **1.07 Any psychotropic prescription, regardless of mental health-related visits = **1.41 ADHD/stimulants = **1.03 Anti-anxiety = **2.36 Anticonvulsant = **2.08 Antidepressant = **1.52 Antipsychotic = **0.94 Bipolar disorder = **0.71 Any psychotropic prescription for those with previous mental health-related visits = **0.71	
Chrapusta 2014, Other: Poland	Mean = 4.3 years (SD 1.2 years) or 10.4 years (SD 3.1 years) *N* = 120	Anxiety - VAS-A	3, 6, 12, & 18 months		Anxiety around wound treatment is higher in pre-school children when compared to school children and reduced across time (3–18 months). Younger children exhibited more anxiety. 3–7 year old children 3 months: mean = 47.50, SD = 24.26 6 months: mean = 37.92, SD = 24.13 12 months: mean = 42.08, SD = 25.57 18 months: mean = 30.83, SD = 23.38 8–13 year old children 3 months: mea*n* = 20.50, SD = 9.46 6 months: mean = 18.83, SD = 8.65 12 months: mean = 19.17, SD = 7.76 18 months: mean = 15.83, SD = 6.19	Not reported	
DeYoung 2012, Australia	Range = 1–6 years *N* = 130	PTSD, MDD, ADHD, ODD, SAD, and specific phobia modules of the DIPA	1–6 months		High rate of comorbidity (73% of the time) with PTSD at 1 month - more likely to have MDD, ODD, SAD and specific phobia. At 6 months, those with PTSD were more likely to have ADHD, ODD, SAD (in 85% of cases). 18% of children had recovering PTSD, 8% chronic PTSD, 2% delayed-onset PTSD. Post hoc tests indicated that at 1 month, children in the chronic group had significantly more PTSS than the recovery (*p* = .045) and resilient group (*p* < .001), and the recovery group had significantly more PTSS than children in the resilient group (p < .001). There was a significant reduction in PTSS across the 6 months for children in the resilient and recovery groups(p < .001), although children in the recovery group had a significantly higher mean number of symptoms at 6 months in comparison to the resilient group (p < .001). Children in the chronic group did not experience any significant reduction in symptoms (*p* = .725) and were experiencing significantly more PTSS than children in the other two groups at6 months (p < .001).	Rates of mental illness after in kids with PTSD after a 1-month follow-up: ODD = **9.57 SAD = **9.57 Specific phobia = **4.68 ^^ 6 month follow-up: ADHD = **12.55 ODD = **27.62 SAD = **7.39 Additionally, children with a new-onset non-PTSD diagnosis at 6 months had a minimum of on PTSS at 1 month and significantly more PTSS in comparison to children with no new onset disorders at 6 months: d = 1.05 DIPA interaction effect sizes PTSD: n^2^ = .34 MDD: n^2^ = .20 ADHD: n^2^ = .05 ODD: n^2^ = .12 SAD: n^2^ = .15	
DeYoung 2014, Australia	Range = 1–6 years *N* = 116	PTSD and PTSS- DIPA, emotional function CBCL	2 weeks – 6 months		Parent trauma history, child premorbid problems, TBSA, parent distress, parent PTSS all contributed to child PTSD at 6 months (R squared = .49). Premorbid CBCL and psychological functioning and concurrent parental PTSS contributed to higher child PTSS at 6 months.	1 month child PTSS predicting … CBCL: R^2^ = .12 6 month child PTSS predicting … CBCL: R^2^ = .14 PTSS: R^2^ = .55 1 month parent PTSS predicts … Child PTSS: R^2^ = .46 6 month parent PTSS predicts … Child PTSS: R^2^ = .43	
Duke 2018, Australia	Range = 0–17 years N = 11,967	Mental health conditions, self-harm, drug/alcohol abuse	≤32 years		The paediatric cohort with unintentional burns experienced elevated rates of post-burn admissions for a psychiatric condition at a rate of 2.6 times higher than the comparison uninjured cohort. Examination of burn severity (TBSA) found post-burn MH admission rates were at least twice as high (adjusted IRR between 2.00 and 2.81) for both severe and minor classifications when compared with the uninjured. While significantly elevated post-burn MH rates were found for all age groups, children between the age of 10 and 15 years at the burn admission experienced the highest admission rate at almost five times higher than that for uninjured children.	Not reported	
Enlow 2019, United States	Range = 7–17 years *N* = 46	PTSS - CRIES, coping - Child Coping Strategies Checklist	> 1 month		Caregiver anxiety and months since injury predicted child PTSS and coping. Caregiver depression also predicted child PTSS and coping. Child mean PTSD score (CRIES) = 18.95 (SD = 17.04)	Hierarchical regression of youth coping and caregiver anxiety predicting youth PTSS: Step 1 Active: R^2^ = .07 Avoidance: R^2^ = .07 Distraction: R^2^ = .07 Social support: R^2^ = .07 Step 2: Active: R^2^ = .20 Avoidance: R^2^ = .31 Distraction: R^2^ = .15 Social support: R^2^ = .20 Step 3: Active: R^2^ = .22 Avoidance: R^2^ = .35 Distraction: R^2^ = .22 Social support: R^2^ = .19	
Goodhew 2014, Australia	Range = 0–18 (mean = 4 years) *N* = 272	Psychiatric morbidity - WHO Composite International Diagnostic Interview version 2, burn-related distress - Impact of Events Scale, suicidality - Mini International Neuropsychiatric Interview, the impact of burn and current self-perceived level of disfiguration	21–31 years		44% had some sort of distress in relation to burn, 11% had reported lifetime suicide attempts, 42% met criteria for at least one DSM-IV disorder in their lifetime and 19% in the past month, 30% had met criteria for depression, 28% had met criteria for anxiety, most prevalent disorder was MSS (15%) and PTSD (12%). Females more likely than males to meet DSM criteria, and visible burn predictive of a DSM diagnosis. Distress predicted by perceived disfigurement and more days spent in hospital.	Predictors of any lifetime DSM disorder Relationship status: **.30 Gender: **1.12 Perceived disfigurement: **.56 Visible burn: **.85 Number of surgical procedures: **.61 Days hospitalised: **.56 Predictors of lifetime PTSD Relationship status: **.35 Gender: **.65 Perceived disfigurement: **.57 Visible burn: **.71 Number of surgical procedures: **.53 Days hospitalised: **.54 Any lifetime depressive disorder Relationship status: **.38 Gender: **.87 Perceived disfigurement: **.55 Visible burn: **1.08 Number of surgical procedures: **.63 Days hospitalised: **.56 Any lifetime anxiety disorder Relationship status: **.27 Gender: **1.08 Perceived disfigurement: **.56 Visible burn: **.70 Number of surgical procedures: **.72 Days hospitalised: **.54	
Graf 2011, Other: Switzerland	Range = 9–48 months *N* = 76	PTSD - PTSD semi-structured interview, emotional problems - CBCL	X = 15 months. Range: 3–48 months)		13.2% of infants/toddlers met full criteria for PTSD, 73.7% met criteria for re-experiencing, 64.5% for avoidance and emotional numbing, 19.7% for increased arousal. CBCL scores were lower than norms in this group. Trauma severity, maternal PTSD and quality of family relations related to PTSD symptoms.	Behaviour problems in children older than 18 months CBCL total score: d = −.33 CBCL internalising score: d = −.25 CBCL externalising score: d = −.41	
Haag, 2017 Switzerland	Range = 1–4 years *N* = 138 (additionally, 138 mothers and 128 fathers)	Acute posttraumatic stress - DSM 5, Young Child PTSD Checklist	None		11.7% of children met full criteria for acute PTSD, while 15.3% met criteria for acute subsyndromal acute PTSD, 66.7% met criteria for intrusion, and 35% met criteria for avoidance and negative alterations in cognition and arousal. Maternal acute stress was significantly associated with higher acute stress in the child (*p* = .03)	Not reported	
Laitakari 2015, Other: Finland	Mean = 7.1 years (SD = 1.4) *N* = 44	Health-related QoL - measured via 17D questionnaire developed for children aged 8–11 years	X = 6.3 years. Range = 5–9 years.		Perceived and expressed health-related QoL in the burned was comparable, or even better (.968) than that of the control population (.936). Among the study cohort, we noted a small but statistically significant difference between boys and girls, as girls fared better on the learning dimension.	Not reported	
Maskell 2013, Australiax	Range = 8–17 years *N* = 66	QoL - The Paediatric Quality of Life Inventory, psychological adjustment and psychopathology - SDQ, self-concept - P-H SCS	Not explicitly reported. Appears to be about 7 years.		Children with burns have lower health-related QoL (except for physical health), and higher emotional conduct, peer, prosocial problems and hyperactivity, than norms. No differences in self-concept. Burned cohort QoL mean 78.87, SD = 15.10 Normative cohort QoL mean = 83.91, SD = 12.47	Not reported	
Murphy 2015, United States	Range = 16–21.5 years (2.5–12.5 years post-burn) Mean = 8.3 years (SD = 3) *N* = 50	Level of disability and function - WHODAS, QoL - BSHS-B	2.5–12.5 years		No difference in 2.5–7.5 and 7.5–12.5 years post-burn functioning. Increased TBSA related to increased disability (except for cognition and self-care). Burns sustained after school entry (older) were associated with more disability.	Not reported	
Nelson 2020, United States	Range = 6–16 years post-burn Mean = 11.97 years (SD = 3.11) *N* = 65	Depressive symptoms, anxiety -PROMISE pediatric profile, PTSD - Child PTSD symptom scale, PTG - Post-traumatic Growth Inventory for Children-Revised	6- and 12-months post-discharge from initial injury		A significant association between pain interference and intensity and depression and anxiety but not PTSD or PTG.	Time 1 with pain interference as the independent (predictor) variable Pain intensity: R^2^ = .36 Physical functioning mobility: R^2^ = .18 Time 1 with pain intensity as the independent (predictor) variable Physical functioning mobility: R^2^ = .23	
Nodoushani 2018, United States	Range = 5–18 years *N* = 836	Physical/psychosocial recovery - BOQ, Depressive symptoms or suicidal ideation - study-specific questionnaire	3–36 months post-discharge		Around 50% of patients maintained a ‘positive’ response from each successive time point. From baseline to 36 months, 45% of children had a ‘positive’ response for at least one-time point, while only 2% had a ‘positive’ response at all time points. Over all 7 time points, the majority (72%) of children with a ‘positive’ response were rated as being depressed ‘Some of the time’ and only a small percentage (4%) of patients with a ‘positive’ response were rated as being depressed ‘All of the time’. The prevalence of these symptoms is greatest at baseline and decreases until it tapers off around 12% at 2 to 3 years.	Not reported	
Rimmer 2014, United States x	Range = 8–17 years *N* = 63	Anxiety - SCARED	Not reported		Panic disorder/significant somatic symptoms (4.9, SD = 4.8), GAD (5.2, SD = 4.3), Separation anxiety disorder (4.2, SD = 3.9), Social anxiety disorder (5.0, SD = 3.6), Significant school avoidance (1.8, SD = 1.8), total (21.1, SD = 14.7). Children reported significantly higher scores than their parents on all subscales. The largest (for social anxiety disorder) indicated that parent and youth report shared only 16% of their variance.	Not reported	
Rimmer 2014b, United States x	Range = 8–17 years *N* = 197	Anxiety - SCARED	Appears to be a mean of about 6 years, given mean age at time of burn was 5.8 and mean current age was 12.4		Seventy-seven participants (39%) screened positive for the possible presence of an anxiety disorder with total SCARED scores of at least 25; 55 (28%) scored 30 or more. A total score of 30 or greater is more specific to the likely presence of anxiety disorder. Nearly half of the youth, 87 (44%), had mean scores 5 on the Separation Anxiety subscale, indicating the presence of separation anxiety symptoms, whereas 55 survivors (28%) had mean scores of 7 on the Panic subscale, indicating the presence of panic disorder or significant somatic symptoms. Finally, 55 youth (28%) had mean scores of 7 on the School Avoidance subscale, indicating the presence of significant school avoidance, 47 (24%) had mean scores of 8 on the Social Anxiety subscale, indicating the presence of social anxiety disorder, and 23% had mean scores of 9 on the Generalized Anxiety Disorder subscale, indicating the presence of generalized anxiety disorder. Significant sex differences were observed for anxiety and somatic complaints, with girls scoring higher in total anxiety and on each anxiety subscale including generalized anxiety, separation anxiety, social phobia, and school phobia.	Not reported	
Riobueno-Naylor 2020, United States	Range = 12–17 Mean = 14.84 years (SD = 1.92) *N* = 78	Psychosocial functioning – PSC-17, social functioning and anxiety – SAS-A, the Perceived Stigmatization Questionnaire, Harter’s SPPA, the Body Image Life Engagement Questionnaire	X = 4.54 years		Prevalence of general psychosocial problems within the participant sample as measured by the PSC-17 overall risk score was 15.3%. Participants who reported appearance concerns had significantly more severe symptoms related to fear of negative evaluation on the SAS-A (Mdn = 17.00) compared with those who did not report appearance concerns (Mdn = 11.50; U = 302.50, *P* < .01). Reports of appearance concerns were also significantly associated with lower ratings of self-worth on the SPPA (Mdn = 3.20) compared with participants who did not report appearance concerns (Mdn = 3.60, U = 277.00, P < .01). Results indicated that the overwhelming majority (70.0%) of adolescents receiving follow-up care for burn injuries reported appearance concerns. No significant differences in median scores were found when comparing scores on measures of social functioning and anxiety across groups based on participant burn size, location, or sex.	Not reported	
Rosenberg 2015, United States	Mean = 15.5 years (SD = 4.6) *N* = 67	DSM diagnosis, effective and cognitive difficulties (physician’s records, neurology consults, psychology records, and psychiatric records from the initial acute hospitalization- Clinical assessment, Acute Stress Disorder Checklist, Impact of Events Scale, CDI, BDI, CBCL	X = 2.8 years		Acute presentation: No differences between EI and control patients in GAD (45 and 52%), depression (19 and 12%), grief (34 and 36%), but there was a difference in ASD/PTSD (51 and 31%) 10% of EI had cognitive difficulties vs. 1% of controls A minority and equal number of the patients in both groups had documented behavioural problems (7 and 9%) Follow-up: GAD (14 and 10%), PTSD (5 and 7%), depression (19 and 13%), grief (14 and 23%), cognitive difficulties (10 and 3%), behaviour problems (7 and 10%).	Not reported	
Russell 2013, United States	Range = 18–28 years (time of burn < 18 years) *N* = 82	Self-concept - Tennessee Self-Concept Scale, 2nd edition, psychiatric illness - Structured Clinical Interview for DSM-IV Disorders, emotion problems - Young Adult Self-Report	> 2 years		Lower self-esteem than norms. As self-concept scores diminished, emotional and behavioral problem scores increased. Participants diagnosed with current anxiety disorders had significantly lower scores for the total self-concept scale, (*P* = .0017), the personal scale (*P* = .0004), and each of the three supplementary scales (identity, *P* = .0018; satisfaction, P = .0018; and behavior, P = .0004). Participants diagnosed with current anxiety disorders had significantly lower scores for the total self-concept scale, (P = .0017), the personal scale (P = .0004), and each of the three supplementary scales (identity, P = .0018; satisfaction, P = .0018; and behavior, P = .0004).	Not reported	
Sveen 2012, Other: Sweden	Range = 5–18 years *N* = 144	Emotional health - BOQ, fear and avoidance, psychological health - SDQ	Between 4 and 12 years		Fear-avoidance was positively associated with measures of pain, itch, and parental concern and was negatively associated with appearance, emotional health, and school re-entry. Emotional health = 89, SD = 18. Itching is associated with psychosocial distress.	Not reported	
Thomas 2012, United States	Range = 18–30 years (burn occurred < 16 years old) *N* = 98	Personality disorder symptoms and diagnosis - Structured Clinical Interview for the DSM-IV-TR Axis II Personality Disorders	Not reported		48 participants (49%) met the criteria for diagnosis with one or more personality disorders. Paranoid Personality Disorder was the most frequent diagnosis found overall (19.4%). Passive Aggressive Personality Disorder was the next most frequent diagnosis overall (18.4%) and the most common personality disorder among women (27.5%). Antisocial Personality Disorder was almost as frequent (17.3%) and the most common personality disorder among men (22.4%). Depressive Personality Disorder (11.2%) and Borderline Personality Disorder (9.2%) were the next most frequent diagnoses. Of those meeting criteria for personality disorder, 21 (43.8%) met criteria for two or more personality disorder diagnoses. Women significantly more likely to meet criteria for Borderline, Avoidant, Passive Aggressive, and Depressive Personality Disorders and men significantly more likely to meet criteria for Antisocial Personality Disorder.	Not reported	
Warner 2012, United States	Range = 0–18 years *N* = 678	Psychological status, appearance, satisfaction, compliance, emotional health - BOQ	< 4 years		Patients with facial burns had significantly slower recovery in the domains of upper extremity function, pain, itch, appearance, satisfaction, emotional health, and family disruption (*p* < 0.05). Patients with burns involving greater than or equal to 20%TBSA had slower improvement in upper extremity function, pain, itch, satisfaction, emotional health, family disruption, and parental concern when compared with patients with burns involving less than 20% TBSA.	Not reported	
Weedon 2011, Other: South Africa	Range = 2–12 years *N* = 70	Quality of life -PedsQL	Not reported		Mean quality of life scores one-week post-discharge from the burns unit were 152.63 (SD = 20.41) and 180.87 (SD = 31.31) three months after discharge. A score of 200 is expected for an optimal quality of life in children using the PedsQL. Bathing, helping to pick up own toys, pain, and energy levels in the physical section and an emotional component (worrying) in the psychosocial section explains why there was a shift within the total score. Children sustaining hot water burns scored lower quality of life scores than children sustaining flame burns.	Not reported	
Willebrand 2011, Other: Sweden	Range = 3–18 years *N* = 181	Emotional problems - SDQ, anxiety and depression - Hospital Anxiety and Depression Scale.	0.3–9 years		For Emotional symptoms, Hyperactivity/Inattention and Prosocial behaviour the percentage of caseness ranged from 11 to 14%, which is close to the norm (10%). For Conduct problems, Peer relationship problems and the Total difficulties score the proportion of indicated cases varied between 18 and 20%. For the Impact score, a cut-off of 1 identified 20% as potential cases, while the cut-off at 2 identified 15% as potential cases. Eight children had pre-burn problems with attention/behaviour, two reported depression and one had sleep problems. Age, visible scars, parents HADS scores, fathers education, and the variable change in living arrangements, together explaining 40% of the variance in emotional problems	Not reported	
Cross-Sectional Studies							
Study authors, year, and country	Participants	Study design	Psychological outcomes/measures	Time since burn		Results	
Nicolosi 2013, Other: Brazil	Range = 12–20 years *N* = 63	Depression - BDI, self-esteem - RSE	Not reported	Mean BDI score was 7.63 (SD 8.72), suggesting that, at most, participants were not or only slightly depressed. Participant answers yielded a mean self-esteem score of 8.41 (SD 4.74), showing that, in general, this group has an adequate degree of self-esteem. Average BDI and RSE scores for teenagers who had sustained burns on their head (*n* = 43) versus those who did not (n = 20) were statistically the same (BDI: *P* = 0.26; RSE: *P* = 0.21). The scores also were not statistically significantly different between persons who did (*n* = 38) compared to those who did not (*n* = 25) sustain burns on their hands (BDI: *P* = 0.10; RSE: *P* = 0.28).		Not reported	
Pardo 2010, Other: Spain	Range = 1–17 years *N* = 139	Anxiety - State-Trait Anxiety in Children, Scale for Anxiety Behavior Observation During Hospitalisation, emotion problems - CBCL	Not reported	State anxiety higher than norms (35.88, SD = 7.85), trait lower than norms (33.93, SD = 6.46), CBCL total scores higher than norms (internalizing and externalizing syndromes (10.52, SD = 6.174; 14.14, SD = 9.139, respectively) and also in the following sub-scales, anxious/depressed (5.70, SD = 3.414), rule-breaking, and aggressive behavior (4.82,SD = 3.872; 9.32, SD = 5.790, respectively)). “boys experience more anxiety (T = 2.011, *P* = .047), while it can also be seen that flames are the cause of significantly higher levels of anxiety in these children (T = 2.089, *P* = .040), higher averages correspond to older children (F = 4.577, *P* = .016; F = 3.710, *P* = .033; and F = 4.990, *P* = .012). CBCL- these scales are significant in relationship to the gender variable (T = 2.128, P = .040; T = 2.237, *P* = .031) in that boys are those who show some kind of behavioral anomaly associated with attention and belligerent behavior”.		Not reported	
Quezada 2016, Other: Mexico	Range = 7–18 years *N* = 51	Resilience - the Resilience Questionnaire for Children and Adolescents	6 years	According to the clinical cut-off points established for the resilience questionnaire for children and adolescents, patients showed a high level of resilience (mean = 128.31, SD = 21.63) with scores ranging between 105 and 157. The variable with the largest effect on the resilience of patients was age at the time of burn, followed by intrusion symptoms of caregiver		Not reported	
Other Studies							
Study authors, year, and country	Participants	Study design	Psychological outcomes/measures	Time since burn	Results	Effect size	
Brown 2014, Australia	Range = 4–13 years *N* = 77	Other: a longitudinal study on data collected through RCT	Anxiety - VAS-A, Child Trauma - CTSQ	None	Higher SAA during wound change procedures associated with higher scores on CTSQ (for 1 point increase on CTSQ SAA increased by 6.79 U/ml (CI = 1.79, 11.78, *p* = .008), mean Child Trauma Screening Questionnaire (CTSQ) at 3 months post-injury was 3.47 (SD = 2.45). More stress during wound dressing associated with higher PTSD symptoms at 3 months post injury.	Not reported	
Conn 2017, United States	Range = 6–12 years *N* = 40	Non-randomised experimental study	Somatic and cognitive anxiety - yoga evaluation questionnaire	Not reported	Somatic and cognitive anxiety were lower after the intervention. Mean somatic anxiety = 2.7 (SD 2.2), mean cognitive anxiety = 2.9 (SD 2.2)	Somatic anxiety: d = .77 Cognitive anxiety: d = .76	
Khadra 2018, Canada	Range = 9 months - 2.4 years *N* = 15	Other: quasi-experimental pilot study	Anxiety - modified Smith Scale, Procedural Behavior Checklist	None	Average level of anxiety was low and no difference across time periods. Percentage of population experiencing anxiety levels: Anxiety level 0 = 53.3% Anxiety level 1 = 33.3% Anxiety level 2 = 6.7% Anxiety level 3 = 6.7% There was a strong positive correlation between pain and procedural anxiety.	Not reported	
Tropez-Arceneaux 2017, Other: Nicaragua	Range = 12–25 years *N* = 33	Non-randomised experimental study	Self-esteem - RSE, self-concept, socializing skills, depression - BDI, CDI, anxiety - Beck Anxiety Scale	5 years	Decrease in anxiety, depression and parent depression, and increase in self-esteem. anxiety = 32, self-esteem = 32, depression = 29	Not reported	

Note: Visual Analog Scale-Anxiety (VAS-A), Child Trauma Screening Questionnaire (CTSQ), Quality of life (QoL), Child Depression Inventory (CDI), State-Trait Anxiety Inventory (STAI/STAIC), Piers-Harris Self-Concept Scale (P-H SCS), Strengths and Difficulties Scale (SDQ), The Paediatric Quality of Life Inventory (PedsQL), Young Child PTSD Checklist (YCPC), Post Traumatic Stress Disorder (PTSD), Randomised controlled trial (RCT), Acute Stress Disorder (ASD)

Note: Visual Analog Scale – Anxiety (VAS-A), adjusted Risk Ratio (aRR), Attention Deficit Hyperactivity Disorder (ADHD), Major Depressive Disorder (MDD), Post-Traumatic Stress Disorder (PTSD), Oppositional Defiance Disorder (ODD), Social Anxiety Disorder (SAD), Diagnostic Infant Preschool Assessment (DIPA), Post-Traumatic Stress Symptoms (PTSS), Total Body Surface Area (TBSA), Child Behavior checklist (CBCL), Incidence rate ratio (IRR), Children’s Revised Impact of Event Scale (CRIES), World Health Organisation (WHO), Strengths and Difficulties Questionnaire (SDQ), Piers-Harris Self-Concept Scale (P-H SCS), World Health Organisation Disability Assessment Schedule (WHODAS), Burn Specific Health Scale – Brief Instrument (BSHS-B), Parent Reported Outcomes Measurement Information System (PROMIE), Post-Traumatic Growth (PTG), Burn Outcomes Questionnaire (BOQ), Screen for Anxiety Related Disorders (SCARED), Pediatric Symptom Checklist (PSC-17), Social Anxiety Scale (SAS), Self-perception Profile for Adolescents (SPPA), Diagnostic and Statistical Manual of Mental Disorders (DSM), Children’s Depression Inventory (CDI), Beck Depression Inventory (BDI), General Anxiety Disorder (GAD)

Note: ** Indicates a transformation of the odds ratio reported in the original study by dividing by 1.81, as described in Chinn (2000), Beck Depression Inventory (BDI), Rosenberg’s Self-esteem Scale (RSE)

Note: Visual Analog Scale – Anxiety (VAS-A), Randomised Controlled Trial (RCT), Childhood Trauma Questionnaire (CTQ), salivary alpha-amylase (SAA), Post-Traumatic Stress Disorder (PTSD)

### Results of quantitative studies

#### Anxiety

The most commonly reported psychological outcome in this review was anxiety in children and adolescents following a burn injury [23–37]. The six cohort studies investigating the presence of anxiety following a pediatric burn examined children with burns versus normative data on uninjured children, or how anxiety related to differing individual factors in the child or adolescent population, such as age or gender. One study compared the anxiety of children or adolescents who had been hospitalised for burns for at least 24-h to normative data and found that state (i.e. situational) and not trait (i.e. related to personality) anxiety was higher in children and adolescents who had experienced a burn injury [31]. Another study that explored anxiety and pain found that pain was significantly associated with anxiety in children or adolescents who had been discharged from hospital for a burn 6–12 months prior [30]. One study investigating age of the child at injury found that age was significantly related to anxiety, with pre-school children exhibiting more anxiety around wound dressings than older children [25]. Rimmer et al. [34, 35] conducted two studies investigating anxiety in burn-injured children and adolescents; one study examined concordance of parent and child ratings of anxiety, and one study investigating anxiety levels generally. Children self-reported their anxiety levels as significantly higher than proxy-reports by their parents [35]. A high proportion of children and adolescents screened positively for anxiety symptoms (39%), with 28% of the population scoring above the cut-off to indicate an anxiety disorder. This is compared to 6.9% of Australian children in the general population [34, 38]. This study also observed that girls with a burn injury scored higher on anxiety measures than boys [35]. Riobueno-Naylor et al. [36] found that children who reported concerns with their appearance after burn were more likely to have social anxiety compared to those who had no appearance concerns, regardless of burn size, bodily location of burn or gender of the child.

There were nine RCTs that investigated anxiety and pediatric burns [23, 24, 26–29, 32, 33, 37]. Three of the studies used the Visual Analog Scale – Anxiety (VAS-A), which has not been validated in a control group for children, however baseline anxiety was comparable for all children with burn injuries in all three studies (mean = 2.6–2.76 [23]; mean = 2.7–2.9 [24]; mean = 2.0 [27]). One of the studies used a measure of anxiety that was created specifically for the intervention (Yoga Evaluation Questionnaire), and thus is not able to be compared to normative data [26]. Khadra et al. [29] found that children’s anxiety (indexed on the Procedural Behaviour Checklist [39]) was low at baseline, but was strongly positively related to procedural pain and fear. Jeffs et al. [28] reported that the children in their study at baseline scored an average of 31.5 (state) and 34 (trait) on the State-Trait Anxiety Inventory [40], which is just below the clinical cut-off for an anxiety disorder [39, 41]. Conversely, Parlak-Gurol et al. [32] reported much higher baseline scores (range = 46.71–45.96) for state anxiety using the same measure, placing their cohort in the clinical range. One study found that children recovering from a burn injury had a baseline score of 32 on the Beck Anxiety Inventory [42], which indicates severe anxiety [37]. Finally, another RCT reported that the children in their study scored at baseline much higher on the Spence Children’s Anxiety Scale [43] than what is reported in normative studies (77.4–90.4, compared to 21.72, respectively; [33]). In summary, anxiety was consistently identified as a common outcome of pediatric burns. This relationship was replicated across multiple studies of differing methodological quality, with age, gender, perceived body-image, procedural pain, and fear noted as influential factors.

#### Traumatic stress

Another common psychological outcome investigated in the literature was traumatic stress or post-traumatic stress symptoms [24, 30, 44–50]. One study found that 8% of children required treatment for Acute Stress Disorder after their burn injury [49]. Another study examining PTSD symptoms found that 11.7% of children met full criteria for PTSD, and a further 15–66.7% met subclinical thresholds for at least one cluster of symptoms (i.e. re-experiencing the event, avoidance, emotional numbing or increased arousal) [48]. Similarly, Graf et al. [47] found that 13.2% of toddlers in their study (aged 9–48 months) met full criteria for PTSD, and between 19.7–73.7% met criteria for at least one cluster of PTSD symptoms. This study also found that the burn severity and quality of family relations were all associated with toddler PTSD symptoms [47]. Several of the studies also found that maternal or parental distress and PTSD symptoms were associated with child PTSD or traumatic stress [45–48]. Brown et al. [44] showed that the stress the child experienced during wound dressings were associated with higher PTSD symptoms 3 months post-injury. De Young et al. [45] found that parental trauma history, child premorbid problems, and burn severity and size were related to child PTSD symptoms 6 months after the injury. In contrast, Nelson et al. [30] found no significant associations between pain and intensity of the burn and PTSD symptoms. Finally, Stoddard et al. [50] found that PTSD symptoms decreased gradually over time for children who had experienced a burn.

#### Depression/mood disturbances

Eight studies investigated depression symptoms in children after a burn injury [30, 33, 37, 50–53]. Boles et al. [51] found that 32% of their sample of children who had experienced a frostbite burn had symptoms of depression at the time of the injury. Nodoushani et al. [52] found similarly high rates of depressive symptoms in their study, with around 45% of participants indicating they felt depressed at some point in the 36 months after their injury, although these results were found using a study-specific questionnaire that has not been validated. Three studies used the Child Depression Inventory [54] to assess depressive symptoms in their samples. The first found scores to exceed the clinical cut-off for depression immediately after hospitalisation [33], with similar high scores in a the same sample 5 years post-burn injury [37]. In contrast, the third study showcased scores that were below the clinical cut-off during hospitalisation [50]. Another study by Nicolosi et al. [55] found that mean depression scores in their sample indicated that most participants were not depressed or displayed only a few symptoms of depression post-burn injury, regardless of where the burn was situated on the body. In contrast, Rosenberg et al. [53] found that 19% of patients with electrical burns had a diagnosis of depression 3 years post-injury. Finally, Nelson et al. [30] found that the pain and intensity of a burn was associated with depression 6- and 12-months post-burn injury.

#### Emotional issues

Eight studies investigated emotional issues, mostly measured via the Child Behaviour Checklist (CBCL) [31, 47, 53, 56], the Strengths and Difficulties Questionnaire [57–59], and the Burn Outcomes Questionnaire (BOQ) [60, 61]. Of the studies using the CBCL, one study found that the children displayed a higher proportion of emotional problems than a normative sample [31], while another found their sample displayed fewer emotional problems than a normative one [47]. A third study using the CBCL reported that 7–9% of participants displayed emotional and behavioural problems [53], however, their results were not compared with normative data. Of the two studies that used the Strengths and Difficulties Questionnaire, one reported comparable results to normative data [59] whereas another study found their sample displayed more emotional problems than normative data [58]. Both studies reporting on the BOQ found significant associations between burns and emotional health [60, 61]. Sveen et al. [60]) identified that pain, itch, and parental concern were associated with fear-avoidance and emotional health, and Warner et al. [61] found that larger burn sizes and facial burns decelerated the pace of emotional recovery [60]. Russell et al. [62] investigated the relationship between self-concept (perception of themselves and subsequent self-esteem) and emotional issues, and found that poorer self-concept was associated with emotional problems.

#### Self-esteem

Findings were mixed with regards to self-esteem in children and adolescents who had experienced a burn injury. Three studies reported that their sample had healthy levels of self-esteem that were comparable to normative or control data of non-burned children [37, 55, 58], whereas two studies reported low self-esteem in their sample [36, 62]. Russell et al. [62] reported that children with an anxiety disorder also had lower self-concept. Riobueno-Naylor et al. [36] found that appearance concerns were associated with lower self-worth, and that the majority of adolescents receiving follow-up care after a burn were concerned with their appearance, although this wasn’t related to burn size, location or gender of the adolescent.

#### Quality of life

Four studies examined Quality of Life (QoL) [58, 63–65]. Laitakari et al. [63] found that QoL was comparable for children who had experienced a burn injury when compared to control groups of non-burned children. Maskell et al. [58] and Weedon and Potterton [65] both found that QoL was lower for children or adolescents who had experienced a burn injury, with the latter study showing that children who were burned with hot water scored the poorest in terms of QoL. Murphy et al. [64] did not compare QoL to normative data, however did note that QoL 2.5–12.5 years after the injury was poorer for children who sustained the injury after school entry (i.e. they were older).

#### Self-harm

One study investigated the incidence of self-harm following a burn injury and found that 10–26 years after the injury, 2.7% of individuals who had experienced a burn injury had been admitted to hospital for self-harm, which is more than double the number of admissions from the non-burned control cohort [66].

#### Suicidality

Goodhew et al. [67] found that in their sample of adults who had sustained a burn injury as children, 11% had reported a suicide attempt in their lifetime. This study indicates individuals who sustained a burn in childhood have higher rates of suicide attempts compared to the general population, which is estimated to be between 3.2–4.5% [68, 69].

#### Mental health diagnoses

Five studies reported on the incidence of mental health diagnoses following a pediatric burn injury [53, 66, 67, 70–72]. Bushroe et al. [70] reported that young children (0–4 years) were most likely to receive a mental health diagnosis (8.56 risk-ratio) compared to older children (10–14 years; 1.02 risk-ratio). Duke et al. [66] found that burn-injured pediatric patients were 2.6 times more likely to be admitted to hospital for psychiatric conditions 10–26 years after the injury, regardless of burn size or severity. This study also found that age was a factor, with older children (10–15 years) being five times more likely to be admitted than younger children. Goodhew et al. [67] reported that 42% of their cohort received a mental health diagnosis in their lifetime, with female gender and burn visibility increasing this risk ratio. Thomas et al. [72] reported that 49% of their sample met criteria for one or more Cluster A (25.5%), Cluster B (31.6%), Cluster C (21.5%), or Other (35.7%; Personality Not Otherwise Specified, Passive Aggressive, or Depressive) personality disorders post a severe paediatric burn injury. Rosenberg et al. [53] investigated diagnosis based on burn type (electrical injury versus other burns) and found comparable incidences of mental health diagnoses at the acute stage post-injury for electrical injury compared to other burns. Specifically, they found that at acute presentation, 45–52% of the sample were experiencing anxiety, 31–51% were experiencing PTSD or Acute Stress Disorder, and 12–19% were experiencing depression. Two-years post-injury these rates dropped to 10–14% for anxiety, 5–7% for PTSD, and 13–19% for depression. Finally, De Young et al. [72] found that the children or adolescents with diagnosed PTSD after they had experienced a burn injury were more likely to also have Major Depressive Disorder, Oppositional Defiance Disorder, Seasonal Affective Disorder and Specific Phobia 1 month post-injury (73% of cases). Six months post-injury, the children with PTSD were more likely to have an additional diagnosis of Attention Deficit Hyperactivity Disorder, Oppositional Defiance Disorder and Seasonal Affective Disorder (85% of cases).

#### Post-traumatic growth, resilience and coping

Two studies examined post-traumatic growth (i.e. positive psychological change), resilience or coping in children and adolescents who had experienced a burn injury [30, 73]. Nelson et al. [30] reported that pain from burns did not interrupt post-traumatic growth in children. Quezada et al. [73] showed that the children in their study had generally high levels of resilience following a burn injury, and that age was significantly associated with resilience, with younger children showing more resilience (as measured via the Resilience Questionnaire for children and adolescents) than older children.

### Results of qualitative studies

Two of the three qualitative studies investigated the experiences of children or adolescents who experienced a burn, and the third investigated parent perception of their child or adolescent following a burn (see Table 2; [72–74]). Themes that arose in all studies were that the child experienced significant anxiety, stress, or “worry” after the burn incident.

**Table 2:** Results of the qualitative studies

Study authors, year, and country	Participants	Inclusion/exclusion criteria	Study design	Psychological outcomes/measures	Time since burn	Results
Coombes 2020, Australia	Range = < 16 years *N* = 18 families (59 family members)	**Inclusion criteria:** Indigenous families with children younger than 16 years old. Children had sustained a burn injury.	Qualitative, Cohort study	Qualitative experiences of trauma	2–3 years.	Participants felt that there was no recognition or treatment for the psychological effects resulting from the injury for the child, or the one who caused the injury, or the family. A family experienced trauma through the removal of a sibling of the injured child to welfare other families were traumatised by fear of their child being removed.
Egberts, 2018 The Netherlands	Range = 12–17 years *N* = 8	**Inclusion criteria**: children had been hospitalized for a burn injury in one of the three Dutch burn centers for a minimum of 24 h and had undergone at least one wound care procedure.	Qualitative cohort study	Qualitative reflection on injury, hospitalisation and their coping.	7 months.	Participants reported three overarching themes; vivid memories, importance of parental support, and psychosocial impact and coping. Children’s vivid memories included experiencing the accident, the look of the wounds and scars, pain, and positive memories of their hospital experience. Children often preferred their parent’s presence because it made them feel safe, although children also reported parental presence was not always necessary or parents did not have to be present all the time. The majority of children reported to have adjusted well to the injury, however there were two type of concerns; concern it might happen again and the reactions of others. And finally, children found it helpful to process the trauma by gradual exposure and putting the injury into perspective. They also reported focussing on positive outcomes. Some children also reported avoiding places and objects that reminded them of the accident.
Horridge 2010, United Kingdom	Range = 8–15 years *N* = 9	**Inclusion criteria:** Parents with children who have experienced burns.	Qualitative cohort study	Parent perception of child psychological functioning	X = 681 days Range: 339–893 days	Parents reported that seeing their children respond to their injury with stress reactions, including disturbed sleep, heightened anxiety, and depression.
McGarry 2014, Australia	Range = 8–15 years *N* = 12	**Inclusion criteria:** Children who sustained a burn injury which required surgery. All children had been patients at PCH.	Qualitative cohort study	Ongoing recurrent trauma; returning to normal activities; behavioural changes; scarring- the permanent reminder	6 months	Themes; the initial trauma: sustaining the burn (All children described feeling “scared”, “worried” and “afraid” at the time of the accident. Children feared they would “die” or “lose a limb”, ongoing paediatric medical trauma (medical events causing further trauma included surgery and dressing changes), return to normal activities (children felt a loss of independence, catastrophic thoughts, upset and disappointed they couldn’t participate in sport), scarring - the permanent reminder (children not wanting to look at or touch their scars, only showing scars to trusted friends, being happy to see improvement in scars, being disappointed they had to wear pressure garments). Children describe experiencing symptoms of acute stress disorder, avoidance, hypervigilance, internalising symptoms, and positive adaption.

### Risk of bias within studies

Based on our criteria (see Table 3), we found that six independent studies met a high level of reliability and quality (58,63,64,66,70,77). Only those six studies used a non-burn comparison group with which to compare psychological outcomes. Fourteen studies reported on longitudinal outcomes (36, 37, 51–53, 55, 60–64, 66, 67, 73), whereas the others reported on outcomes during the first 18 months following the injury.

**Table 3:** Risk of bias and study quality assessment

Study	Non-burn comparison group?	Validated measure or confirmed diagnosis	Power analysis	Appropriate analysis	Prospective or longitudinal design (> 18mo)	Total
Boles, 2018	0	1	0	1	1	3
Brown, 2014	0	1	1	1	0	3
Brown, 2014	0	1	1	1	0	3
Bushroe, 2018	1	1	0	1	0	4
Chester, 2018	0	1	1	1	0	3
Chrapusta, 2014	0	1	0	1	0	2
Conn, 2017	0	0	1	1	0	2
DeYoung, 2012	0	1	0	1	0	2
DeYoung, 2014	0	1	0	1	0	2
Duke, 2018	1	1	0	1	1	4
Enlow, 2019	0	1	1	1	0	3
Goodhew, 2014	0	1	0	1	1	3
Graf, 2011	0	1	0	1	0	2
Haag, 2017	0	1	0	1	0	3
Hyland, 2015	0	1	1	1	0	3
Jeffs, 2014	0	1	1	1	0	3
Khadra, 2018	0	1	1	1	0	3
Laitakari, 2015	1	1	0	1	1	4
Maskell, 2013, 2014	1	1	1	1	0	4
Murphy, 2015	1	1	0	1	1	4
Nelson, 2020	0	1	0	1	0	2
Nicolosi, 2013	0	1	0	1	1	3
Nodoushani, 2018	0	0	0	0	1	1
Pardo, 2010	0	1	0	1	0	2
Parlak Gurol, 2010	0	1	0	1	0	2
Quezada, 2016	0	1	0	1	1	3
Rezazadeh, 2020	0	1	1	1	0	3
Rimmer, 2014a/b	0	1	0	1	0	2
Riobueno-Naylor, 2020	0	1	0	1	1	3
Rosenberg, 2015	0	1	0	1	1	3
Russell, 2013	0	1	0	1	1	3
Sharp, 2010	0	1	1	1	0	3
Stoddard, 2011	0	1	0	1	0	2
Sveen, 2012	0	1	0	1	1	3
Thomas, 2012	0	1	0	1	0	2
Tropez-Arceneaux, 2017	0	1	0	0	1	2
Warner, 2012	0	1	0	1	1	3
Weedon, 2011	0	1	0	1	0	3
Willebrand, 2011	0	1	0	1	0	2

Note: 1 = present, 0 = not present

Due to the heterogeneity of study designs, we were unable to conduct a meta-analysis on whether children and adolescents who experienced a burn injury went on to experience elevated rates or risk of psychopathology later in life.

## General discussion

This study aimed to examine the research conducted on the psychological impact on children’s mental health following paediatric burn injury from the years 2010–2020. In line with our hypothesis, we found that most studies reported elevated levels of psychopathology and psychological symptoms following paediatric burn injuries. However, findings were mixed for most mental health concerns and kinds of symptoms, except for increased anxiety symptoms and traumatic stress post burn injury.

Regarding psychological symptoms, studies reported that children generally experience more anxiety following a burn injury, that the anxiety they experience is more likely to be state-based (than trait-based), and the anxiety following a burn is associated with pain, the age at which the burn occurred (older children exhibit more difficulties), and the gender of the child (with girls more likely to report experiencing more anxiety than boys) [28, 30–37]. Qualitatively, we found that parents and children/adolescents both report the child or adolescent experiencing heightened anxiety after a burn compared to prior [75, 76, 78]. The majority of studies that investigated traumatic stress following a burn injury also reported children experiencing higher PTSD and acute stress symptoms, and that these symptoms could also be related to parental distress and the child’s premorbid psychological problems [45, 47–49, 71].

In terms of mental health diagnoses, the six studies which reported these had different designs, making it difficult to generalise their results [53, 66, 67, 70–72]. There does appear to be evidence, however, of elevated risk for mental health diagnoses and hospitalisation following paediatric burn injury, and in particular diagnoses such as Anxiety Disorders, PTSD, Acute Stress Disorder, Depression, and Personality Disorders were reported by the studies to be higher for this population.

Findings were mixed regarding depressive symptoms, emotional issues, self-esteem and QoL [30, 47, 51, 58–65, 77], and thus we cannot conclude on the experiences of children following a burn in these mental health concerns. Further, there were few studies investigating self-harm, resilience and suicidality following paediatric burn injury [66, 67, 73], and further evidence is required for conclusions to be drawn in these domains.

## Limitations and future considerations

There were methodological strengths and issues that arose from the studies in this review. Impressively, we found that 88% of the studies in our review used standardised measures, an improvement in comparison to the review by Bakker et al. [10], who reported that just 75% of included studies used standardised measures. This increase in the use of standardised measures allows for more reliable and generalisable findings. One of the main limitations we found was the issue of heterogeneity meaning we were unable to conduct a meta-analysis. Some studies reported lifetime diagnoses, and did not differentiate whether the diagnosis was pre- or post-burn injury [67], and other studies did not report on factors that would likely influence outcomes (such as whether the children received psychological support after the injury) [53]. Standardised reporting of mental health outcomes, for instance routinely collecting comorbid mental health diagnoses at admission or collecting standardised measures of depression and anxiety for all burns patients, would help address the issue of heterogeneity across studies. Further, more studies are needed in terms of long-term diagnostic outcomes of paediatric burns patients. The paper that reported the strongest evidence for long-term elevated rates of psychopathology was that of Bushroe et al. [70] who examined risk ratios for mental health diagnoses longitudinally and provided clear indication of the long-term impact of a paediatric burn injury. Most studies in our review reported on short-term (acute-18 months) psychological outcomes following a burn injury, and it is difficult to determine causality in such studies. Despite this, we believe that the findings are still useful and able to inform clinical care and practice.

## Conclusions

Given that most of the participants in the studies experienced increased anxiety, and that many experienced other psychological symptoms following a burn injury, it is clear that children and adolescents are particularly vulnerable following this type of traumatic injury. The longitudinal studies included in this review demonstrate that risk for psychopathology following a paediatric burn injury is much higher and ongoing than in the general population, which suggests that psychological recovery for these children and adolescents needs to be an area of focus in the future. The clinical care teams involved in a child’s recovery from a burn need to support psychological recovery for the child and the family to promote optimal outcomes. Ideally, the clinical care team should include consultation from mental health professionals. Our findings also demonstrate that factors in a child’s life (such as parental distress, age, and gender) may influence their psychological recovery and should be taken into consideration by the clinical care team.

## Data Availability

Data was available to authors via University databases. The datasets used and/or analysed during the current study available from the corresponding author on reasonable request.

## Abbreviations

PTSD: Post-traumatic Stress Disorder
PRISMA: Preferred Reporting Items for Systematic Reviews and Meta-Analyses
RCT: Randomised Controlled Trial
VAS-A: Visual Analog Scale – Anxiety
CBCL: Child Behaviour Checklist
BOQ: Burns Outcomes Questionnaire
QoL: Quality of Life

## References

[1] Australian Institute of Health and Welfare. Hospitalised burn injuries Australia [Internet]. Australian Institute of Health and Welfare. 2013 [cited 2020 Oct 19]. Available from: https://www.aihw.gov.au/reports/injury/hospitalised-burn-injuries-australia-2013-14/contents/summary

[2] Toon MH, Maybauer DM, Arceneaux LL, Fraser JF, Meyer W, Runge A (2011). Children with burn injuries-assessment of trauma, neglect, violence and abuse. J Inj Violence Res.

[3] Chong HP, Quinn L, Cooksey R, Molony D, Jeeves A, Lodge M (2020). Mortality in paediatric burns at the Women’s and Children’s hospital (WCH), Adelaide, South Australia: 1960–2017. Burns..

[4] Yarbrough DR. Improving survival in the burned patient. J S C Med Assoc 1975. 1990 Jun;86(6):347–9. 2398733

[5] De Sousa A (2010). Psychological aspects of Paediatric burns (a clinical review). Ann Burns Fire Disasters.

[6] Martin-Herz SP, Thurber CA, Patterson DR (2000). Psychological principles of burn wound pain in children II: treatment applications. J Burn Care Rehabil..

[7] Andreasen NJ, Noyes R, Hartford CE (1972). Factors influencing adjustment of burn patients during hospitalization. Psychosom Med.

[8] Andreasen NJ, Hartford CE, Knott JR, Canter DA (1977). EEG changes associated with burn delirium. Dis Nerv Syst.

[9] Van Loey NEE, Van Son MJM (2003). Psychopathology and psychological problems in patients with burn scars: epidemiology and management. Am J Clin Dermatol.

[10] Bakker A, Maertens KJP, Van Son MJM, Van Loey NEE (2013). Psychological consequences of pediatric burns from a child and family perspective: a review of the empirical literature. Clin Psychol Rev.

[11] Dise-Lewis JE (2001). A Developmental Perspective on Psychological Principles of Burn Care: J Burn Care Rehabil.

[12] Landolt MA, Buehlmann C, Maag T, Schiestl C (2020). Brief report: quality of life is impaired in pediatric burn survivors with posttraumatic stress disorder. J Pediatr Psychol.

[13] Saxe GN, Stoddard FJ, Hall E, Chawla N, Lopez C, Sheridan R, et al. Pathways to PTSD, part I: children with burns. Am J Psychiatry. 2005;6. 10.1176/appi.ajp.162.7.1299 15994712

[14] Young ACD, Kenardy JA, Cobham VE, Kimble R. Prevalence, comorbidity and course of trauma reactions in young burn-injured children. J Child Psychol Psychiatry. 2011;8. 10.1111/j.1469-7610.2011.02431.x 21671940

[15] Stoddard FJ, Ronfeldt H, Kagan J, Drake JE, Snidman N, Murphy JM, et al. Young burned children: the course of acute stress and physiological and behavioral responses. Am J Psychiatry. 2006;7. 10.1176/ajp.2006.163.6.1084 16741210

[16] Meyer WJ, Robert R, Murphy L, Blakeney PE (2000). Evaluating the psychosocial adjustment of 2- and 3-year-old pediatric burn survivors. J Burn Care Rehabil.

[17] Piazza-Waggoner C, Dotson C, Adams CD, Joseph K, Goldfarb IW, Slater H (2005). Preinjury Behavioral and Emotional Problems Among Pediatric Burn Patients. J Burn Care Rehabil.

[18] Stoddard FJ, Norman DK, Murphy MJ, Beardslee WR (1989). Psychiatric outcome of burned children and adolescents. J Am Acad Child Adolesc Psychiatry.

[19] Landolt MA, Grubenmann S, Meuli M (2002). Family Impact Greatest: Predictors of Quality of Life and Psychological Adjustment in Pediatric Burn Survivors. J Trauma Inj Infect Crit Care.

[20] Liberati A, Altman DG, Tetzlaff J, Mulrow C, Gøtzsche PC, Ioannidis JPA (2009). The PRISMA statement for reporting systematic reviews and meta-analyses of studies that evaluate health care interventions: explanation and elaboration. J Clin Epidemiol.

[21] Veritas health innovation. Covidence systematic review software [Internet]. Covidence. [cited 2020 Oct 22]. Available from: https://app.covidence.org/

[22] National Heart Lung and Blood Institute. Study Quality Assessment Tools: Study quality assessment tool for observational cohort and cross-sectional studies [Internet]. : https://www.nhlbi.nih.gov/health-topics/study-qualityassessment-tools. 38. Abrutyn S, Mueller AS. 2020 [cited 2020 Oct 22]. Available from: https://www.nhlbi.nih.gov/health-topics/study-quality-assessment-tools

[23] Brown NJ, Kimble RM, Rodger S, Ware RS, Cuttle L (2014). Play and heal: randomized controlled trial of ditto™ intervention efficacy on improving re-epithelialization in pediatric burns. Burns..

[24] Chester SJ, Tyack Z, De Young A, Kipping B, Griffin B, Stockton K (2018). Efficacy of hypnosis on pain, wound-healing, anxiety, and stress in children with acute burn injuries: a randomized controlled trial. Pain..

[25] Chrapusta A, Pąchalska M (2014). Evaluation of differences in health-related quality of life during the treatment of post-burn scars in pre-school and school children. Ann Agric Environ Med.

[26] Conn AS, Hall MS, Quinn K, Wiggins B, Memmott C, Brusseau TA (2017). An examination of a yoga intervention with pediatric burn survivors. J Burn Care Res..

[27] Hyland EJ, D’Cruz R, Harvey JG, Moir J, Parkinson C, Holland AJA (2015). An assessment of early child life therapy pain and anxiety management: a prospective randomised controlled trial. Burns..

[28] Jeffs D, Dorman D, Brown S, Files A, Graves T, Kirk E (2014). Effect of Virtual Reality on Adolescent Pain During Burn Wound Care: J Burn Care Res.

[29] Khadra C, Ballard A, Déry J, Paquin D, Fortin J-S, Perreault I (2018). Projector-based virtual reality dome environment for procedural pain and anxiety in young children with burn injuries: a pilot study. J Pain Res.

[30] Nelson S, Uhl K, Wright LA, Logan D (2020). Pain is associated with increased physical and psychosocial impairment in youth with a history of burn injuries. J Pain.

[31] Pardo GD, García IM, Gómez-Cía T (2010). Psychological Effects Observed in Child Burn Patients During the Acute Phase of Hospitalization and Comparison With Pediatric Patients Awaiting Surgery. J Burn Care Res.

[32] Parlak Gürol A, Polat S, Nuran AM (2010). Itching, Pain, and Anxiety Levels Are Reduced With Massage Therapy in Burned Adolescents. J Burn Care Res.

[33] Rezazadeh H, Froutan R, Ahmad Abadi A, Mazloum SR, Moghaddam K (2020). Effects of art therapy program on anxiety and depression among 6-12-year-old burned children. Open Access Maced J Med Sci.

[34] Rimmer RB, Bay RC, Alam NB, Sadler IJ, Hansen L, Foster KN, et al. Burn-Injured Youth May Be at Increased Risk for Long-Term Anxiety Disorders: J Burn Care Res 2014;35(2):154–161. 10.1097/BCR.0b013e31828c73ac 24165666

[35] Rimmer RB, Bay RC, Sadler IJ, Alam NB, Foster KN, Caruso DM (2014). Parent vs burn-injured child self-report: contributions to a better understanding of anxiety levels. J Burn Care Res..

[36] Riobueno-Naylor A, Williamson H, Canenguez K, Kogosov A, Drexler A, Sadeq F, et al. Appearance Concerns, Psychosocial Outcomes, and the Feasibility of Implementing an Online Intervention for Adolescents Receiving Outpatient Burn Care. J Burn Care Res. 2020 Jun 28;iraa108. 10.1093/jbcr/iraa108 32594128

[37] Tropez-Arceneaux LL, Castillo Alaniz AT, Lucia Icaza I, Alejandra Murillo E. The Psychological Impact of First Burn Camp in Nicaragua: J Burn Care Res 2017;38(1):e1–e7. 10.1097/BCR.0000000000000465 27893579

[38] AIHW. Australia’s children, Children with mental illness [Internet]. Australian Institute of Health and Welfare. 2020 [cited 2021 Feb 3]. Available from: https://www.aihw.gov.au/reports/children-youth/australias-children/contents/health/children-with-mental-illness

[39] Elliott CH, Jay SM, Woody P (1987). An observation scale for measuring children’s distress during medical procedures. J Pediatr Psychol.

[40] Knight RG, Waal-Manning HJ, Spears GF (1983). Some norms and reliability data for the state--trait anxiety inventory and the Zung self-rating depression scale. Br J Clin Psychol.

[41] Julian LJ. Measures of Anxiety. Arthritis Care Res [Internet]. 2011 Nov [cited 2021 Feb 3];63(0 11). Available from: https://www.ncbi.nlm.nih.gov/pmc/articles/PMC3879951/10.1002/acr.20561 PMC3879951 22588767

[42] Beck AT, Epstein N, Brown G, Steer RA (1988). An inventory for measuring clinical anxiety: psychometric properties. J Consult Clin Psychol.

[43] Spence MJ, Moore DS (2003). Categorization of infant-directed speech: development from 4 to 6 months. Dev Psychobiol.

[44] Brown NJ, Kimble RM, Rodger S, Ware RS, McWhinney BC, Ungerer JPJ (2014). Biological markers of stress in pediatric acute burn injury. Burns..

[45] De Young AC, Hendrikz J (2014). Kenardy J.a. JA, Cobham VE, Kimble RM. prospective evaluation of parent distress following pediatric burns and identification of risk factors for young child and parent posttraumatic stress disorder. J Child Adolesc Psychopharmacol.

[46] Enlow PT, Brown Kirschman KJ, Mentrikoski J, Szabo MM, Butz C, Aballay AM (2019). The role of youth coping strategies and caregiver psychopathology in predicting posttraumatic stress symptoms in pediatric burn survivors. J Burn Care Res..

[47] Graf A, Schiestl C, Landolt MA (2011). Posttraumatic stress and behavior problems in infants and toddlers with burns. J Pediatr Psychol.

[48] Haag A-C, Landolt MA (2017). Young Children’s acute stress after a burn injury: disentangling the role of injury severity and parental acute stress. J Pediatr Psychol.

[49] Sharp S, Thomas C, Rosenberg L, Rosenberg M, Meyer W (2010). Propranolol Does Not Reduce Risk for Acute Stress Disorder in Pediatric Burn Trauma. J Trauma Inj Infect Crit Care.

[50] Stoddard FJ (2017). Therapeutic play in the diagnosis and treatment of hospitalized children recovering from acute burns. J Am Acad Child Adolesc Psychiatry.

[51] Boles R, Gawaziuk JP, Cristall N, Logsetty S (2018). Pediatric frostbite: a 10-year single-center retrospective study. Burns..

[52] Nodoushani A.Y., Murphy J.M., Wang S.L., Stoddard FJ, Kazis L., Lydon M., et al. Prevalence of depressive symptoms over time in pediatric burn survivors. J Burn Care Res. 2018;39(Supplement 1):S102.

[53] Rosenberg M, Mehta N, Rosenberg L, Ramirez M, Meyer WJ, Herndon DN (2015). Immediate and long-term psychological problems for survivors of severe pediatric electrical injury. Burns..

[54] Kovacs M (2011). Children’s depression inventory.

[55] Nicolosi JT, De Carvalho VF, Sabates AL (2013). A quantitative, cross-sectional study of depression and self-esteem in teenage and young adult burn victims in rehabilitation. Ostomy Wound Manag.

[56] Achenbach TM, Rescorla LA (2013). Manual for the ASEBA School-age Forms & Profiles.

[57] Goodman R (2001). Psychometric properties of the strengths and difficulties questionnaire. J Am Acad Child Adolesc Psychiatry.

[58] Maskell J, Newcombe P, Martin G, Kimble R. Psychosocial Functioning Differences in Pediatric Burn Survivors Compared With Healthy Norms: J Burn Care Res 2013;34(4):465–476. 10.1097/BCR.0b013e31827217a9 23702857

[59] Willebrand M, Sveen J, Ramklint M, Bergquist M, Huss F, Sjöberg F (2011). Psychological problems in children with burns—parents’ reports on the strengths and difficulties questionnaire. Burns..

[60] Sveen J, Huss F, Sjoberg F, Willebrand M (2012). Psychometric properties of the swedish version of the burn outcomes questionnaire for children aged 5 to 18 years. J Burn Care Res..

[61] Warner P, Stubbs TK, Kagan RJ, Herndon DN, Palmieri TL, Kazis LE (2012). The effects of facial burns on health outcomes in children aged 5 to 18 years: J trauma acute care Surg. Sep.

[62] Russell W, Robert RS, Thomas CR, Holzer CE, Blakeney P, Meyer WJ (2013). Self-Perceptions of Young Adults Who Survived Severe Childhood Burn Injury: J Burn Care Res.

[63] Laitakari E, Koljonen V, Pyorala S, Rintala R, Roine RP, Sintonen H (2015). The long-term health-related quality of life in children treated for burns as infants 5-9 years earlier. Burns..

[64] Murphy ME, Holzer CE, Richardson LM, Epperson K, Ojeda S, Martinez EM (2015). Quality of life of young adult survivors of pediatric burns using world health organization disability assessment scale II and burn specific health scale-brief: a comparison. J Burn Care Res.

[65] Weedon M, Potterton J (2011). Socio-economic and clinical factors predictive of paediatric quality of life post burn. Burns..

[66] Duke JM, Randall SM, Vetrichevvel TP, McGarry S, Boyd JH, Rea S, et al. Long-term mental health outcomes after unintentional burns sustained during childhood: a retrospective cohort study. Burns Trauma. 2018 Dec 1;6:s41038–018-0134-z. 10.1186/s41038-018-0134-z PMC6233288 30460320

[67] Goodhew F, Van Hooff M, Sparnon A, Roberts R, Baur J, Saccone EJ (2014). Psychiatric outcomes amongst adult survivors of childhood burns. Burns..

[68] Johnston AK, Pirkis JE, Burgess PM (2009). Suicidal thoughts and Behaviours among Australian adults: findings from the 2007 National Survey of mental health and wellbeing. Aust N Z J Psychiatry.

[69] De Leo D, Cerin E, Spathonis K, Burgis S (2005). Lifetime risk of suicide ideation and attempts in an Australian community: prevalence, suicidal process, and help-seeking behaviour. J Affect Disord.

[70] Bushroe KM, Hade EM, McCarthy TA, Bridge JA, Leonard JC. Mental Health after Unintentional Injury in a Pediatric Managed-Medicaid Population. J Pediatr. 2018 Aug;199:29–34.e16. 10.1016/j.jpeds.2018.03.039 29747938

[71] De Young AC, Kenardy JA, Cobham VE, Kimble R (2012). Prevalence, comorbidity and course of trauma reactions in young burn-injured children. J Child Psychol Psychiatry.

[72] Thomas CR, Russell W, Robert RS, Holzer CE, Blakeney P, Meyer WJ (2012). Personality disorders in Young adult survivors of pediatric burn injury. J Personal Disord.

[73] Quezada L, González MT, Mecott GA (2016). Explanatory Model of Resilience in Pediatric Burn Survivors: J Burn Care Res.

[74] Coombes J, Hunter K, Mackean T, Ivers R (2020). The journey of aftercare for Australia’s first nations families whose child had sustained a burn injury: a qualitative study. BMC Health Serv Res.

[75] Horridge G, Cohen K, Gaskell S (2010). BurnEd: parental, psychological and social factors influencing a burn-injured child’s return to education. Burns..

[76] McGarry S, Elliott C, McDonald A, Valentine J, Wood F, Girdler S (2014). Paediatric burns: from the voice of the child. Burns..

[77] Maskell J, Newcombe P, Martin G, Kimble R (2014). Psychological and psychosocial functioning of children with burn scarring using cosmetic camouflage: a multi-Centre prospective randomised controlled trial. Burns..

[78] Egberts MR, van de Schoot R, Geenen R, Van Loey NEE (2017). Parents’ posttraumatic stress after burns in their school-aged child: a prospective study. Health Psychol.

